# A Novel Retrovirus (Gunnison’s Prairie Dog Retrovirus) Associated With Thymic Lymphoma in Gunnison’s Prairie Dogs in Colorado, USA

**DOI:** 10.3390/v12060606

**Published:** 2020-06-02

**Authors:** Molly D. Butler, Karen Griffin, Connie D. Brewster, Marylee L. Kapuscinski, Mark D. Stenglein, Daniel W. Tripp, Sandra L. Quackenbush, Karen A. Fox

**Affiliations:** 1 Department of Microbiology, Immunology and Pathology, College of Veterinary Medicine and Biomedical Sciences, Colorado State University, Fort Collins, CO 80523, USA; molly.butler@colostate.edu (M.D.B.); conniebrew@gmail.com (C.D.B.); mllayton@rams.colostate.edu (M.L.K.); mark.stenglein@colostate.edu (M.D.S.); 2 Colorado Division of Parks and Wildlife, Wildlife Health Laboratory, Fort Collins, CO 80521, USA; karen.griffin@state.co.us (K.G.); dan.tripp@state.co.us (D.W.T.)

**Keywords:** *Cynomys gunnisoni*, endogenous retrovirus, prairie dog, retrovirus, thymic lymphoma, tumor, *Yersinia pestis*

## Abstract

As part of research and wildlife disease surveillance efforts, we performed necropsy examinations of 125 free-ranging (*n* = 114) and captive (*n* = 11) prairie dogs in Colorado from 2009 to 2017. From these cases, we identified three cases of thymic lymphoma in free-ranging Gunnison’s prairie dogs (*Cynomys gunnisoni*), and we identified a novel retroviral sequence associated with these tumors. The viral sequence is 7700 nucleotides in length and exhibits a genetic organization that is consistent with the characteristics of a type D betaretrovirus. The proposed name of this virus is Gunnison’s prairie dog retrovirus (GPDRV). We screened all 125 prairie dogs for the presence of GPDRV using PCR with envelope-specific primers and DNA extracted from spleen samples. Samples were from Gunnison’s prairie dogs (*n* = 59), black-tailed prairie dogs (*Cynomys ludovicianus*) (*n* = 40), and white-tailed prairie dogs (*Cynomys leucurus*) (*n* = 26). We identified GPDRV in a total of 7/125 (5.6%) samples including all three of the prairie dogs with thymic lymphoma, as well as spleen from an additional four Gunnison’s prairie dogs with no tumors recognized at necropsy. None of the GPDRV-negative Gunnison’s prairie dogs had thymic lymphomas. We also identified a related, apparently endogenous retroviral sequence in all prairie dog samples. These results suggest that GPDRV infection may lead to development of thymic lymphoma in Gunnison’s prairie dogs.

## 1. Introduction

Colorado is home to three of the five free-ranging prairie dog species native to North America: the white-tailed prairie dog (*Cynomys leucurus*), the black-tailed prairie dog (*Cynomys ludovicianus*), and the Gunnison’s prairie dog (*Cynomys gunnisoni*). Prairie dogs are a member of the *Sciuridae* family, along with squirrels and other burrowing rodents. Prairie dogs are considered “keystone” species in that prairie dogs and their burrows provide prey and habitat for a number of wild birds and mammals, they support diverse plant and pollinator communities and the health of the local ecosystem often depends on the health of prairie dog populations [1,2]. Disease threats from plague (caused by *Yersinia pestis*) are well understood in prairie dogs [3,4], which are highly susceptible to fatal infections from *Y. pestis*. However, the roles of other infectious agents in the health of free-ranging prairie dog colonies have not been extensively considered.

Much of the available information about prairie dog health, including the incidence of cancer, comes from studies in prairie dogs that are kept in captivity either as pets or for research. The most commonly reported tumors of captive prairie dogs are elodontoma and hepatocellular adenocarcinoma, of which hepatocellular carcinoma may be associated with infection by a hepadnavirus [5,6]. Other tumors are less commonly encountered in captive prairie dogs but do include lymphoma and thymoma [5,7,8]. An infectious cause for lymphoid tumors in prairie dogs has not been investigated, although retroviruses are a well-known cause of several human and veterinary lymphoid cancers [9,10,11,12,13,14,15]. In some cases, such as infection with feline leukemia virus and feline immunodeficiency virus, possible sequela of retroviral infection also include immune suppression [16,17,18,19,20]. 

During the course of wildlife disease research and surveillance activities in Colorado, USA, we identified three free-ranging Gunnison’s prairie dogs with thymic lymphoma, and a novel type D betaretrovirus associated with these tumors. Based on the known sensitivity of prairie dogs to sylvatic plague, we also considered retroviral infections as a possible source of immune suppression that could contribute to mortality.

## 2. Materials and Methods 

### 2.1. Necropsy, Histopathology, and Immunohistochemistry

From 2009 to 2017, we necropsied 125 free-ranging (*n* = 114) and captive (*n* = 11) prairie dogs from Colorado. Species examined included Gunnison’s (*n* = 59), black-tailed (*n* = 40), and white-tailed (*n* = 26) prairie dogs. Animals examined were either found dead (*n* = 118), died during processing (*n* = 3), or were euthanized due to disease concerns (*n* = 4). Research methods included trapping and brief anesthesia with isoflurane gas [21] and were approved (06/06/2013) by the Colorado Parks and Wildlife Animal Care and Use Committee #06-2013.

Gross necropsy was performed for all 125 prairie dogs to determine cause of death, and we pursued histopathology if cause of death was not apparent from gross necropsy and tissues were suitable. Prior to necropsy, the carcasses were either frozen at –20 °C to preserve the carcass and to kill fleas, or fresh carcasses were treated with insecticide (Deltamethrin/DeltaDust, Bayer Environmental Science, Cary, NC, USA) prior to necropsy to kill fleas without the need for freezing. Necropsies were conducted in a biological safety cabinet (NU-S813-400, Nuaire, Plymouth, MN, USA) with additional personal protective equipment in accordance with BSL-2 biosafety practices. After necropsy, carcasses were frozen at –20 °C until *Y. pestis* PCR results were obtained, and any carcasses with tissues confirmed positive for *Y. pestis* or *Francisella tularensis* were disposed of by chemical digestion. For histopathology, tissues were fixed in 10% neutral buffered formalin, embedded in paraffin wax, sectioned by microtome to approximately 8 micrometers, affixed to glass slides, and stained with hematoxylin and eosin.

We used immunohistochemistry to identify T-lymphocytes (CD-3 (LN10, Leica Biosystems, Buffalo Grove, IL, USA)), B-lymphocytes (PAX-5 (1EW, Leica Biosystems, Buffalo Grove, IL, USA)), and epithelial cells/cytokeratin (MCK (AE1/AE3, Leica Biosystems, Buffalo Grove, IL, USA)) in formalin-fixed paraffin embedded tissues. The above monoclonal mouse anti-human antibodies were applied using a Leica BOND-MAX automated IHC staining platform (Leica Biosystems, Buffalo Grove, IL, USA), with chromogen Poly-AP anti-mouse (PV6110, PowerVision, Leica Biosystems, Buffalo Grove, IL, USA) used for PAX-5 and MCF, and chromogen Poly-HRP anti-mouse (PV6113, PowerVision, Leica Biosystems, Buffalo Grove, IL, USA) used for CD-3. Slides were counterstained with hematoxylin. Negative controls of duplicate tissue sections were incubated in antibody diluent and homologous nonimmune sera. Non-specific staining was not observed. To confirm efficacy in prairie dog tissues, we applied IHC stains to control tissues, including thymus, spleen, and skin from a yearling prairie dog that died from enteric disease. These control tissues demonstrated expected staining properties including robust staining of T-lymphocytes in the thymus with CD-3, scattered staining of B-lymphocytes in the thymus with PAX-5, and staining of epithelial cords and nests (Hassall’s corpuscles) in the thymus with MCK. Control prairie dog spleen demonstrated robust staining with PAX-5, highlighting follicular structure, and skin epithelium demonstrated robust staining with MCK. 

### 2.2. PCR for Yersinia Pestis and Francisella Tularensis

We extracted DNA from spleen tissue of all (*n* = 125) prairie dogs, under BSL-2 conditions, using a DNeasy blood and tissue kit (Qiagen, Valencia, CA, USA). Each sample was tested for presence of *Y. pestis* by PCR using primers *caf*1-F/*caf*1-R (Table 1) [22] and cycling conditions as previously described [23]. Primers (50µM) and DNA template (50–250 ng) were added to a 0.2 mL PCR tube containing a puReTaq Ready-To-Go PCR bead (Illustra, GE Healthcare Bio-Sciences Corp, Piscataway, NJ, USA) for a final volume of 25 µL. Cycling conditions were: 94 °C for 10 min (1 cycle), followed by 94 °C for 1 min, 55 °C for 1 min, 72 °C for 30 s (35 cycles), and 72 °C for 10 min (1 cycle). The final product was visualized on a 2% agarose gel. DNA extracted from spleen (see above) was also tested for *F. tularensis* using primers P2/P3 (Table 1) and cycling conditions as previously described [24]. Primers (20 µM) and DNA template (50–250 ng) were added to a 0.2 mL PCR tube containing a puReTaq Ready-To-Go PCR bead (Illustra, GE Healthcare Bio-Sciences Corp, Piscataway, NJ, USA) for a final volume of 25 µL. Cycling conditions were: 97 °C for 10 min (1 cycle), followed by 94 °C for 1 min, 55 °C for 1 min, 75 °C for 1 min (35 cycles), and 75 °C for 10 min (1 cycle). The final product was visualized on a 2% agarose gel.

### 2.3. RNA Extraction and RT-PCR

Total RNA was extracted from fresh-frozen spleen (case nos. 11-1310, 14-1342, 15-1406) and thymic tumor (case nos. 14-1342, 15-1406) tissue samples using TRIzol (ThermoFisher Scientific, Waltham, MA, USA) according to the manufacturer’s instructions. One microgram of DNased (Ambion Turbo DNase; ThermoFisher Scientific, Waltham, MA, USA) RNA was reverse transcribed into cDNA using SuperScript III (ThermoFisher, Scientific, Waltham, MA, USA) according to the manufacturer’s protocol. A 3:1 mix of random hexamers and oligo-dT or the degenerate primer, YMDD, was used as the reverse primer (Table 1). PCR was performed using *Taq* DNA polymerase (ThermoScientific, Waltham, MA) with the degenerate primers LPQG and YMDD (Table 1). PCR reactions contained 2 µL cDNA in reaction buffer comprised of 200 nM of each primer, 2 mM MgCl_2_, 0.2 mM dNTPs and 0.5 units of Fermentas *Taq* DNA polymerase (ThermoFisher Scientific, Waltham, MA, USA) in a total volume of 50 µL. The cycling conditions were as follows for cycles 1–10: 94 °C for 1 min, 37 °C for 2 min, and 72 °C for 3 min. The conditions for cycles 11–40 were: 94 °C for 30 s, 55 °C for 1 min, and 72 °C for 1 min [25]. The PCR product was purified (QIAquick PCR Purification Kit (Qiagen, Valencia, CA, USA)), cloned into the pCR2.1 TOPO vector (ThermoFisher Scientific, Waltham, MA, USA) and submitted for sequencing.

### 2.4. Library Preparation, NextGen Sequencing, Genome Assembly and Analysis

The KAPA Biosystems RNA HyperPrep Kit (Roche, Pleasanton, CA, USA) was used to prepare sequencing libraries from 100 ng total RNA isolated from the lymphoma and spleen from two Gunnison’s prairie dogs (14-1342, 15-1406) with tumors, the spleen from two Gunnison’s prairie dogs (15-656, 15-671) without tumor, and the spleen from a Black-tailed prairie dog (14-1382) without tumor according to the manufacturer’s protocol using half-scale reactions without fragmentation. Pooled libraries were length-selected for 300–500-bp fragments using a BluePippin 2% cassette (Sage Biosciences, Beverly, MA, USA). Length-selected libraries were cleaned using a 1:1.4 ratio of solid phase reversible immobilization [26] beads (Kapa Biosystems, Roche, Pleasanton, CA, USA). Individual libraries were then pooled for sequencing. The library pool was diluted to 4 nM based on fluorometric DNA quantification (Qubit High Sensitivity DNA Assay; ThermoFisher Scientific, Waltham, MA, USA) and quantified by qPCR using the KAPA Library Quantification Kit (Kapa Biosystems, Roche, Pleasanton, CA, USA). Paired-end 2 × 150 bp sequencing was performed on an Illumina NextSeq, producing an average of 5 × 10^6^ read pairs per dataset. 

Datasets were processed as previously described, with the goal of taxonomically categorizing all non-prairie dog reads [27]. Briefly, first low quality and adapter sequences were filtered using Cutadapt v1.18 [28]. Duplicate read pairs (reads that shared >96% pairwise identity) were removed using cd-hit v4.8.1 [29]. Bowtie2 was used to remove host-derived reads by mapping to a combined index built from the *Marmota marmota* (European marmot; GCF_001458135.1) and *Ictidomys tridecemlineatus* (thirteen-lined ground squirrel; GCF_000236235.1) genomes and transcriptomes [30,31]. Reads with an alignment score >60 were removed. Remaining reads were assembled using the SPAdes assembler [32]. Contigs were taxonomically assigned by searching the NCBI nt database using BLASTN and then by searching the NCBI nr database using diamond [33,34]. Candidate retrovirus-derived contigs were manually inspected in Geneious Prime 2020.0.3 (https://www.geneious.com) and validated by re-mapping reads using bowtie2 as above. This analysis pipeline is available at https://github.com/stenglein-lab/taxonomy_pipeline. Libraries from HeLa cell total RNA and water were constructed and analyzed in parallel as positive and negative controls.

PCR and Sanger sequencing confirmation of the Gunnison’s prairie dog retrovirus (GPDRV) genome assembly was performed on high-molecular weight splenic DNA from a tumor-negative but GPDRV sequence positive prairie dog using a forward primer in the LTR (LTR-F) and a reverse primer in *env* (Env-7073). PCR conditions were as follows: 98 °C for 30 s, then 30 cycles of 98 °C for 10 s, 66 °C for 20 s, and 72 °C for 4min, followed by 72 °C for 5 min. A single PCR product of the expected size (7266 bp) was cloned into Strataclone Blunt vector (Agilent Technologies, LaJolla, CA, USA) Plasmid DNA was isolated from individual colonies. Samples were confirmed positive for the insert by restriction digest and sent for Sanger sequencing. The sequence was confirmed using primer walking. 

Fisher’s exact test was performed as implemented in R [35].

### 2.5. Phylogenetics

To collect relevant Pol sequences, we used two strategies. First, we queried the GPDRV Pol sequence against the NCBI nr protein database using BLASTP [36] and retrieved all aligning sequences that produced alignments with E-values lower than 10^–40^. We removed sequences shorter than 600 amino acids and used cd-hit to collapse sequences that shared >95% pairwise identity [28,29]. Secondly, we collected all Pol sequences in the NCBI RefSeq protein database annotated under the family *Retroviridae* (taxid 11632). To collect Env transmembrane protein (TM) domain sequences, we collected all Env sequences in the NCBI RefSeq protein database annotated under the family *Retroviridae*. Because of the high level of sequence divergence between retrovirus Env sequences, we selected the subset of these Env TM refseqs that produced a blastp alignment with an E-value <10^–40^.

In all of these cases, we aligned collected sequences using the MAFFT aligner v7.407 with default parameters and trimmed alignments using TrimAL v1.4.rev15 [37,38]. The best model for tree inference was selected using modeltest-ng and trees were created using RaxML-ng v. 0.9.0 with standard parameters [39,40]. Trees were visualized using the Interactive Tree Of Life (iTOL) v4 [41].

### 2.6. Integration Site Analysis

We performed an integration site analysis using Retro-X Integration Site Analysis Kit (Clontech, Mountain View, CA, USA) according to the manufacturer’s instructions. Briefly, high molecular weight genomic DNA was isolated from tumor tissue and then digested with restriction enzymes *Ssp*1, *Hpa*1, or *Dra*1. Digested DNA was purified and then ligated to GenomeWalker (Clontech, Mountain View, CA, USA) adaptors using T4 DNA ligase. A primary PCR reaction was performed on the adaptor-ligated DNA using an outer, adaptor-specific forward primer AP1, and an outer, GPDRV sequence-specific reverse primer (SP3, SP4, or SP5, Table 1). A secondary, or nested PCR reaction was performed using the primary PCR reaction amplicons as template, a nested adaptor-specific primer AP2, and a nested PDRV sequence-specific reverse primer (SP3 or LTR-R, Table 1). Secondary PCR products were visualized by electrophoresis on an agarose gel to confirm a single, predominant PCR product. PCR products were cloned (Topo TA (ThermoFisher, Waltham MA, USA)) and plasmid DNA was isolated from individual colonies. Samples were confirmed positive for inserts by restriction digest and submitted for Sanger sequencing. Sequences that partially overlapped on the 3’ end with the 5’ end of the PDRV sequence, but that diverged upstream were identified as integration sites.

### 2.7. PCR Screening for GPDRV and ERV-PDRV.1-Cynomys Ludovicianus

Endogenous pro/pol: Primers 494F/591R (Table 1), were used to screen DNA extracted from forty-two prairie dog spleen samples (DNeasy blood and tissue kit (Qiagen, Valencia, CA, USA)). These samples were selected to include captive (*n* = 7) and free-ranging (*n* = 35) animals representing the timespan of the entire project, and originating from carcasses with minimal to mild autolysis suggesting good DNA quality. DNA template (10 ng) was added to a PCR mixture containing 5 µL 10x *Taq* buffer with (NH4)_2_SO_4_, 0.5 µL Taq polymerase (Fermentas, ThermoFisher Scientific, Waltham MA, USA), 200 µM (each) dNTPs, 500 pmol (each primer), 1 mM MgCl_2_, and sterile water to a final volume of 50 µL. Cycling conditions were: 95 °C for 3 min (1 cycle), followed by 95 °C for 30 s, 53.7 °C for 30 s, 72 °C for 30 s (30 cycles), and 72 °C for 5 min. The final product was visualized on a 1% agarose gel.

GPDRV gag gene: Primers 393F/1961R (Table 1), were used to screen DNA extracted from forty-two prairie dog spleen samples (DNeasy blood and tissue kit (Qiagen, Valencia, CA, USA)). DNA template (10 ng) was added to a PCR mixture containing 5 µL 10x *Taq* buffer with (NH4)_2_SO_4_, 0.5 µL Taq polymerase (Fermentas, ThermoFisher Scientific, Waltham, MA, USA), 200 µM (each) dNTPs, 500 pmol (each primer), 1 mM MgCl_2_, and sterile water to a final volume of 50 µL. Cycling conditions were: 95 °C for 3 min (1 cycle), followed by 95 °C for 30 s, 51 °C for 45 s, 72 °C for 30 s (30 cycles), and 72 °C for 5 min. The final product was visualized on a 1% agarose gel.

GPDRV env gene: Primers 5729F/5840R (Table 1), were used to screen DNA extracted from prairie dog spleen samples (DNeasy blood and tissue kit (Qiagen, Valencia, CA, USA)). DNA template (10 ng) was added to a PCR mixture containing 5 µL 10× *Taq* buffer with (NH4)_2_SO_4_, 0.5 µL Taq polymerase (Fermentas, ThermoFisher Scientific, Waltham, MA, USA), 200 µM (each) dNTPs, 500 pmol (each primer), 1 mM MgCl_2_, and sterile water to a final volume of 50 µL. Cycling conditions were: 95 °C for 3 min (1 cycle), followed by 95 °C for 30 s, 54.3 °C for 30 s, 72 °C for 30 s (30 cycles), and 72 °C for 5 min. The final product was visualized on a 1% agarose gel.

Primers 5729F/5840R (Table 1) were also used to screen DNA extracted from an additional eighty-three prairie dog spleen samples (DNeasy blood and tissue kit (Qiagen, Valencia, CA, USA)). Primers (0.5 µM) and DNA template (50–250 ng) were added to a 0.2 mL PCR tube containing a puReTaq Ready-To-Go PCR bead (Illustra, GE Healthcare Bio-Sciences Corp, Piscataway, NJ, USA) for a final volume of 25 µL. Cycling conditions were: 95 °C for 3 min (1 cycle), followed by 95 °C for 30 s, 54.3 °C for 30 s, 72 °C for 30 s (30 cycles), and 72 °C for 5 min (1 cycle). The final product was visualized on a 2% agarose gel.

### 2.8. Deposition of Sequences and of Expression Data

Sequences have been deposited in GenBank under accession numbers MT361316, MT361317, and MT316318. The data have been deposited with links to BioProject accession number PRJNA631279 in the NCBI BioProject database (https://www.ncbi.nlm.nih.gov/bioproject/). 

## 3. Results

### 3.1. Thymic Lymphoma in Gunnison’s Prairie Dogs

Causes of death in prairie dogs included bacteremia, trauma, capture-related factors, environmental factors, intraspecific aggression, euthanasia for disease concern, and thymic lymphoma (Table 2). Plague, caused by *Y. pestis*, accounted for nearly all (49/54; 91%) of the cases of bacterial disease. Two prairie dogs had severe lesions of tularemia, caused by *F. tularensis*. Three Gunnison’s prairie dogs were affected by thymic lymphoma. No other tumors were observed in any of the other 122 prairie dogs examined. The three cases of thymic lymphoma are further described below.

Thymic lymphoma case number 11-1310 was a free-ranging yearling female Gunnison’s prairie dog found dead lying half-way out of a burrow with no evidence of trauma. The chest was filled with cloudy fluid and few fibrin strands. The lungs were mottled and the heart was dilated. The thymus was enlarged to approximately 10× normal size by a soft, white mass with a mottled appearance suggesting multifocal hemorrhage and necrosis. Histopathology was complicated by freeze/thaw artifacts but, demonstrated uniform infiltrates of neoplastic lymphocytes determined to be T-cells by immunohistochemistry for CD-3. The tumor did not contain a significant population of B-cells or epithelial cells as determined by immunohistochemistry for Pax5 and MCK. Neoplastic cells invaded beyond the tissue capsule of the mass and infiltrated the surrounding adipose tissue. The tumor was diagnosed as T-cell lymphoma. Metastasis was not observed. The spleen and liver were PCR negative for *Y. pestis* and *F. tularensis*.

**Figure 1:**
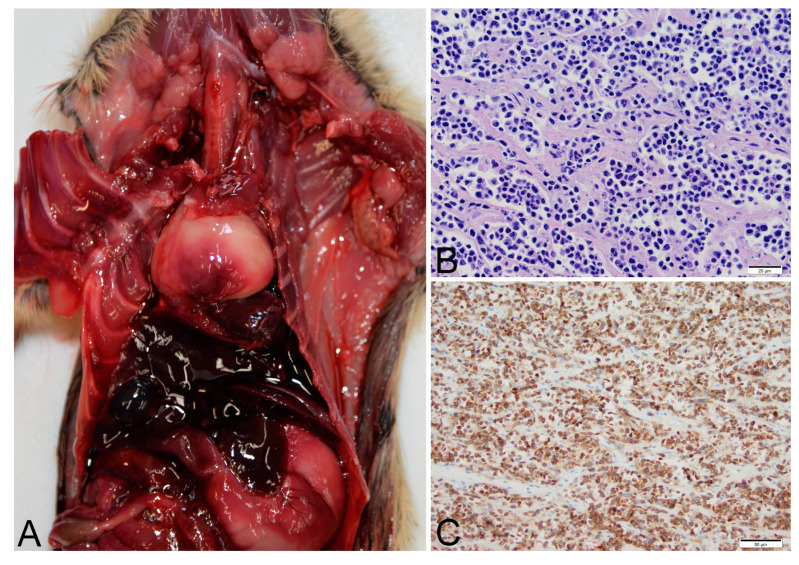
Thymic lymphoma identified in a Gunnison’s prairie dog (Case 14-1342). (**A**) An approximately 3 cm diameter, soft, white mass displaces the heart caudally. (**B**) Histologic findings included uniform infiltrates of neoplastic lymphocytes (hematoxylin and eosin). (**C**) Immunohistochemical staining with anti-CD3 identified neoplastic cells as T-lymphocytes.

Thymic lymphoma case number 14-1342 was a free-ranging, lactating adult female Gunnison’s prairie dog trapped as part of research and management activities. The prairie dog was observed to have slightly labored breathing when found in the trap but did not raise concern for illness. The same prairie dog had been captured approximately one-year prior without complications. While anesthetized, the prairie dog was noted to have stopped breathing. Oxygen was administered but the prairie dog never recovered from anesthesia. At necropsy, erythema of the skin was observed on the vulva and lower limbs. An approximately 1 cm diameter granuloma was present within the mesenteric adipose tissue of the abdomen. Firmly adhered to the base of the trachea was an approximately 3 cm diameter mass that displaced the heart caudally (Figure 1). The mass was white and firm, with a mottled appearance on cut surface suggesting hemorrhage and necrosis. Histopathology was complicated by freeze/thaw artifacts, but demonstrated uniform infiltrates of neoplastic lymphocytes (Figure 1) determined to be T-cells by immunohistochemistry for CD-3 (Figure 1). The tumor did not contain a significant population of B-cells or epithelial cells as determined by immunohistochemistry for Pax5 and MCK. The tumor was diagnosed as T-cell lymphoma. Metastasis was not observed. PCR of liver and spleen were negative for *Y. pestis* and *F. tularensis.*

Thymic lymphoma case number 15-1406 was a free-ranging, approximately 1–2 year old female Gunnison’s prairie dog found dead in a burrow with no evidence of trauma. Body condition was good, with plentiful fat stores in the abdomen. The chest contained an approximately 4 cm diameter mass, with red-tinged fluid filling the chest cavity. The mass was white, soft, and contained multifocal hemorrhages on cut surface. The spleen was moderately enlarged, and the inguinal lymph nodes were dark red. Histopathology was complicated by freeze/thaw artifacts, but the thymic mass demonstrated uniform infiltrates of neoplastic lymphocytes determined to be T-cells by immunohistochemistry for CD-3. The tumor did not contain a significant population of B-cells or epithelial cells as determined by immunohistochemistry for Pax5 and MCK. The tumor was diagnosed as T-cell lymphoma. Metastasis was not observed. PCR of liver and spleen were negative for *Y. pestis* and *F. tularensis*.

### 3.2. Identification of Prairie Dog Retroviral Sequences Associated with Thymic Lymphoma

Initial investigation for a possible retroviral etiology involved use of reverse transcription polymerase chain reaction (RT-PCR) with degenerate retrovirus primers (LPQG and YMDD) targeting a well-conserved region in the reverse transcriptase gene [25]. Sequencing of the PCR products from tumors 14-1342 and 15-1406 identified two unique sequences with homology to known retroviruses.

To obtain additional sequences of these two potential viruses we utilized a meta-genomics approach. Total RNA was isolated from the lymphoma and spleen from two Gunnison’s prairie dogs (14-1342, 15-1406) with tumors, the spleen from two Gunnison’s prairie dogs (15-656, 15-671) without tumor, and the spleen from a black-tailed prairie dog (14-1382) without tumor. An individual library was prepared from each sample and sequenced on an Illumina NextSeq. Sequencing produced an average of 5 × 10^6^ 2 × 150 read pairs per sample. Following removal of low quality and adapter sequences, duplicate reads, and host-derived reads, an average of 2.4 × 10^4^ read pairs remained in each dataset (0.5% of starting datasets). These remaining reads were assembled and the resulting contigs were taxonomically classified.

**Figure 2:**
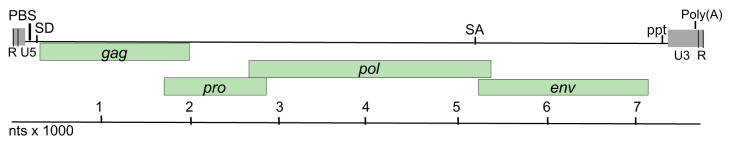
Gunnison’s prairie dog retrovirus genome organization. Diagram of the assembled GPDRV genome from tumors from prairie dogs 14-1342 and 15-1406. The Gag-Pro-Pol polyprotein is predicted to be translated from the genome by ribosomal frameshifting. Location of predicted sites-PBS-primer binding site, SD-splice donor, SA-splice acceptor, ppt-polypurine tract, poly(A)- polyadenylation sequence.

Retroviral sequences were identified in the lymphoma and spleen from the two tumor-positive animals, but not in the spleen from the three tumor negative animals. A complete sequence of 7700 nucleotides (nt) in length was assembled from both tumors. The organization of the assembled genome appeared to be similar to that of betaretroviruses with *gag*, *pro*, *pol* and *env* genes (Figure 2). The nucleotide sequences of the two assembled genomes were 99.2% identical. The two sequences differ at 57 nucleotides resulting in eight amino acid changes in Gag, two in Pro, one in Pol and five in Env. The *pro* and *pol* genes are predicted to utilize ribosomal frameshifting for expression of *gag-pro* and *gag-pro-pol* precursors. The *env* transcript is likely generated by splicing. These data represent the first report of retroviral sequences associated with lymphoma in Gunnison’s prairie dogs, with the provisional name Gunnison’s prairie dog retrovirus (GPDRV).

### 3.3. Prairie Dog Endogenous Retroviral Sequence

A second, distinct retroviral sequence was identified and a consensus contig was assembled from the black-tailed prairie dog (14-1382) spleen sample. This sequence is 6132 nucleotides in length and includes predicted *gag, pro*, and *pol* genes. An *env-*coding region and LTR sequences were not definitively identified. Sequence reads from the tumors and all spleen samples (animals 14-1342, 15-1406, 15-656, 15-671 and 14-1382) align with the 14-1382 consensus contig suggesting this sequence is likely an endogenous retrovirus, provisionally named ERV- PDRV.1-*Cynomys ludovicianus*. This sequence shares 54% nucleotide identity with the GPDRV *gag-pro-pol* sequence.

### 3.4. Features of the Gunnison’s Prairie Dog Retrovirus Sequence, Predicted Proteins and Phylogenetic Analysis

#### 3.4.1. LTR and Untranslated Regions

The long terminal and the 5’ and 3’ untranslated regions of retroviral genomes contain regulatory sequences that are central for viral replication. The LTR of GPDRV is 403 bases in length and is bound by inverted repeat sequences CAAG (nt 94–97) and CTTG (nt 7376–7379) that are essential for integration. U3 is 308 bp, R is 15 bp and U5 is 79 bp (Figure 2). The U3 region is preceded by a polypurine tract (nt 7358–7375), the site for initiation of plus-strand synthesis of viral DNA during retroviral replication. The U3 region in the 5’ LTR serves as the promoter and enhancer for transcription of viral RNA. A consensus TATA box (TATATAA) is located 29 bp upstream of the predicted transcription initiation site. Binding sites for the transcription factors, NF1, AP1, Elk1, and NF-AT are present in the U3 region. The highly conserved polyadenylation signal, AATAAA, is located in the 3’ LTR at nt 7680–7686, similar in position to that of the betaretroviruses, Jaagsiekte sheep retrovirus (JSRV), and enzootic nasal tumor virus (ENTV) [42,43].

The 5’ untranslated region harbors a predicted primer-binding site (PBS) with sequence complementary to the 15 bases at the 3’ end of tRNA^Gln^ that would serve as the site for reverse transcriptase to initiate minus-strand DNA synthesis. A predicted splice donor site for the generation of the subgenomic *env* transcript is located at nt 124–131 in the untranslated region between the PBS and start of *gag*.

#### 3.4.2. Gag

The *gag* open reading frame (nt 212–1915) is predicted to encode a 567 amino acid (aa), 62.8 kDa polyprotein. The *n* terminus of GPDRV Gag contains a consensus myristylation motif (Met-Gly) like that of many retroviruses. The GPDRV capsid protein (CA) is predicted to be a 206 aa, 22.8 kDa protein. A highly conserved major homology region (MHR), QGPSESYSDFIGRLMQSA, is located in CA. Two Cys-His motifs (Cys-X_2_-Cys-X_4_-His-X_4_-Cys) separated by 14 amino acids are located in the nucleocapsid protein (NC) at nt positions 1565–1607 and 1649–1690. The NC protein is predicated to be 14.3 kDa.

#### 3.4.3. Pro

**Figure 3:**
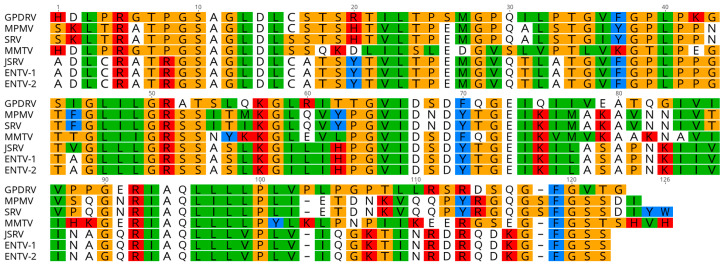
Amino acid alignment of the dUTPase domain from several betaretroviruses. Amino acid alignment was constructed using sequences GPDRV- Gunnison’s prairie dog retrovirus (this manuscript), MPMV- Mason-Pfizer monkey virus (NC_001550), SRV (M11841), MMTV (NC_001503), JSRV (NC_001494), ENTV-1 (NC_007015), and ENTV-2 (NC_00494) with Geneious Prime 2019.2.3.

The *pro* open reading frame (nt 1705–2712) is predicted to encode a 334 aa, 35.7 kDa protein expressed as a Gag-Pro fusion polypeptide generated by ribosomal frameshifting. The protein encoded by the *pro* open reading frame is comprised of two domains, similar to that of betaretroviruses: a pseudoprotease domain with dUTPase activity and the active protease (Figure 3). GPDRV dUTPase exhibits 54–57% amino acid identity with other betaretroviruses. The active protease site with a core aspartyl protease sequence, Leu-Asp-Thr-Gly, is located at amino acid 198-201 (nt 2296–2307). A glycine-rich G patch domain similar in sequence to that found in betaretroviruses is present near C-terminus of protease [44,45].

#### 3.4.4. Pol

The *pol* open reading frame (nt 2685–5375) is predicted to encode a peptide of 896 aa with a molecular mass of 101.8 kDa. Pol is predicted to be expressed as a Gag-Pro-Pol polypeptide generated by ribosomal frameshifting. The Pol polypeptide encodes reverse transcriptase and integrase activity. The conserved polymerase sequences LPQG and YMDD are located at amino acids 157 and 191 (nt 3153 and 3255), respectively. RT contains an RNase H domain with a conserved active site (DEDD). There is an *n*-terminal Zn^+^ binding domain present in the integrase protein.

**Figure 4:**
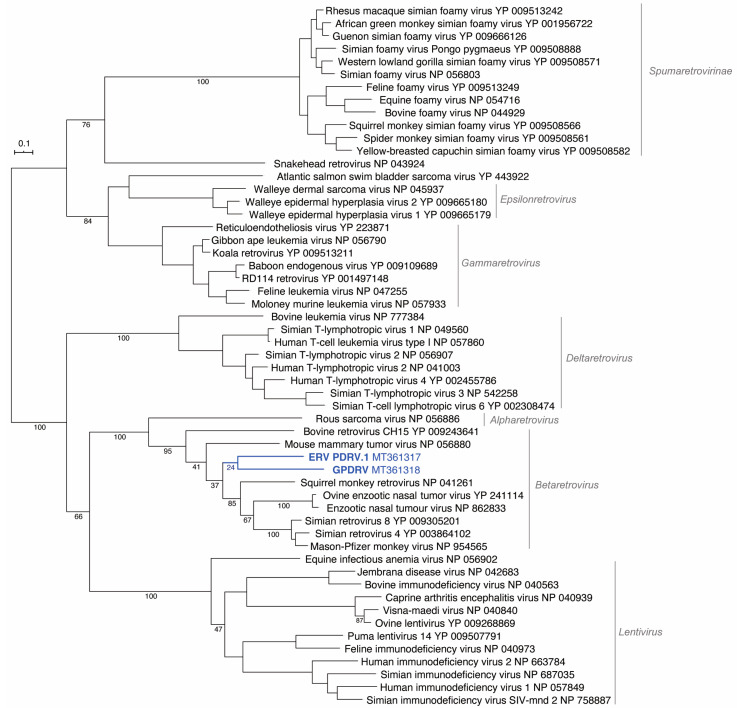
GPDRV is a betaretrovirus. All Pol sequences in the NCBI RefSeq protein database annotated as belonging to the *Retroviridae* family longer than 600 amino acids were used to infer a maximum-likelihood tree. The Felsenstein bootstrap (FBP) support values of select branches are indicated. Retrovirus genera and subfamilies (except for *Spumaretrovirinae*) are indicated. The tree is unrooted and was arbitrary midpoint rooted.

**Figure 5:**
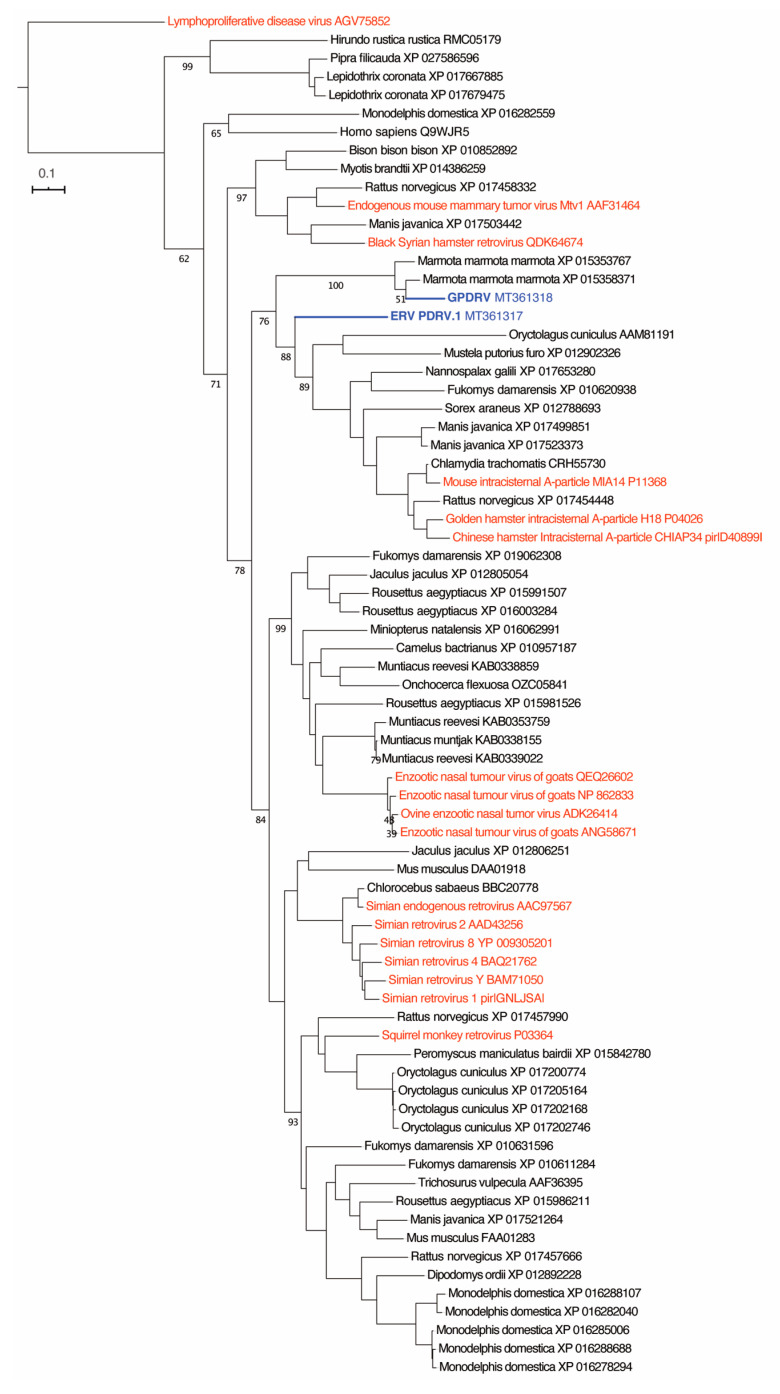
GPDRV is most closely related to retroviral-like sequences in the marmot genome. A tree was made from the sequences in the NCBI protein database most closely related to GPDRV Pol as determined by a BLASTP search. The Felsenstein bootstrap (FBP) support values of select branches are indicated. The GPDRV and GPD ERV Pol sequences are colored blue. Sequences annotated as belonging to the *Retroviridae* family are colored red. The rest of the sequences are annotated as belonging to the indicated mammalian species and are present in the corresponding genome assemblies. The tree was rooted using lymphoproliferative disease virus Pol, which was included as an outgroup.

To determine the relationship between GPDRV and members of established retrovirus subfamilies and genera the entire Pol amino acid sequence was used to infer phylogenies. We took a two-fold approach to identify closely related sequences. First, we created a tree using all of the Pol sequences in the NCBI RefSeq protein database that were annotated as belonging to viruses in the family *Retroviridae* (Figure 4). In this tree, GPDRV and ERV- PDRV.1 clustered within betaretrovirus Pol sequences (genus *Betaretrovirus)* (Figure 4). Second, we used BLASTP to identify the most closely related protein sequences in the NCBI protein database: those producing alignments with E-values less than 10^–40^ (Figure 5). The most closely related sequences were a mixture of retroviral and endogenous retroviral-like sequences from mammalian genome assemblies. The GPDRV Pol sequences clustered with sequences present in the alpine marmot (*Marmota marmota*) genome assembly [30]. In fact, GPDRV Pol was more closely related to these marmot sequences than to ERV- PDRV.1 Pol (Figure 5).

#### 3.4.5. Env

**Figure 6:**
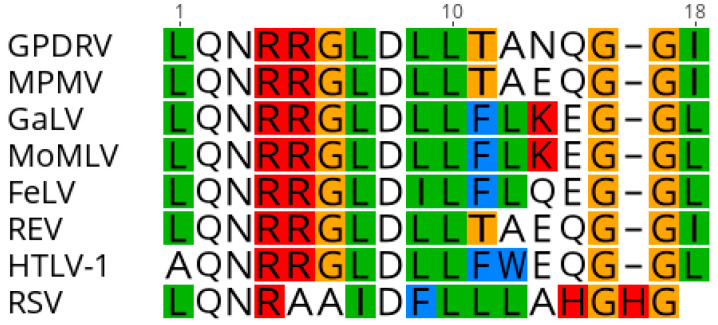
Alignment of the immunosuppressive domain from retroviral transmembrane protein sequences. Amino acid alignment was constructed using sequences GPDRV- Gunnison’s prairie dog retrovirus (this manuscript), MPMV- Mason-Pfizer monkey virus (NC_001550), GaLV- Gibbon ape leukemia virus (NC_001885), MoMLV-Moloney murine leukemia virus (NC_001501), FeLV- feline leukemia virus (NC_001940), REV-reticuloendotheliosis virus NC_006934), HTLV-1- human T cell leukemia virus (NC_001436), and RSV- Rous sarcoma virus (NC_001407) with Geneious Prime 2019.2.3.

**Figure 7:**
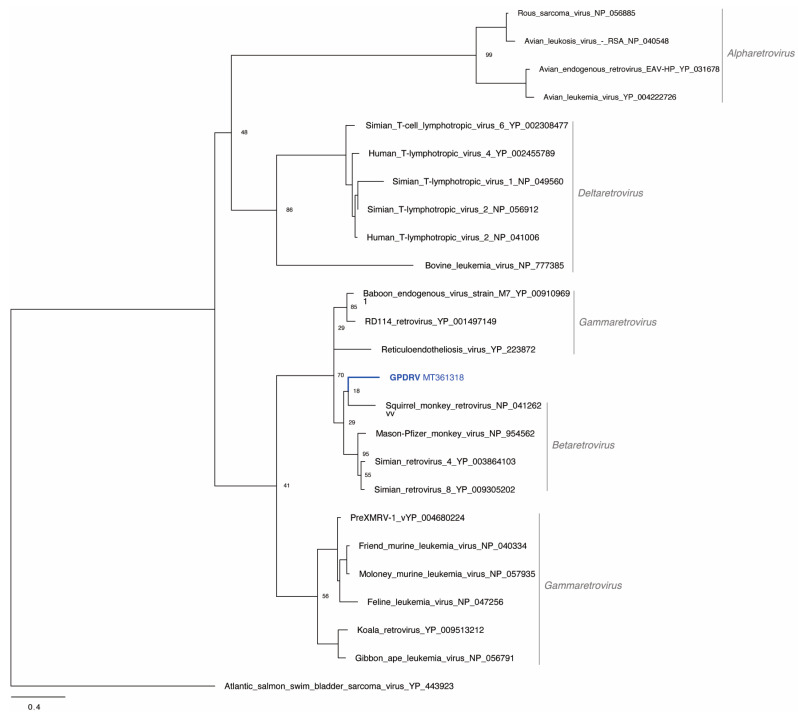
GPDRV Env transmembrane protein (TM) clusters with gamma and betaretrovirus sequences. All ENV TM sequences in the NCBI RefSeq protein database annotated as belonging to the *Retroviridae* family were used to infer a maximum-likelihood tree. The Felsenstein bootstrap (FBP) support values of select branches are indicated. Retrovirus genera are indicated. The tree is unrooted and was arbitrary midpoint rooted.

The envelope protein is likely translated from a spliced transcript that utilizes a splice acceptor site located at nucleotide 5209. The *env* open reading frame (nt 5275–7092) is predicted to encode a 605 amino acid, 64.9 kDa protein. A hydrophobic region located from nt 5275 to 5388 would serve as the signal peptide. Proteolytic cleavage at the furin cleavage consensus recognition site (RHRR) in Env would generate a 410 aa, 43.7 kDa surface protein (SU) and 195 aa, 21.2 kDa transmembrane protein (TM). The TM subunit contains two heptad repeats (HR1 and HR2) that form a coiled coil structure. Located between HR1 and HR2 resides a conserved immunosuppressive domain (ISD) (Figure 6) followed by a cysteine-rich region (CX_6_CC), which is predicted to form a covalent disulfide bond with SU. The GPDRV ISD is 94% identical to that found in Mason-Pfizer monkey virus [46]. A 21 aa hydrophobic region within TM (nt 6850–6945) likely serves as the transmembrane anchor with a 43 amino acid cytoplasmic region. There are ten predicted *n*-linked glycosylation sites, nine in SU and one in TM. Phylogenetic analysis of the TM subunit demonstrates GPDRV TM is found in the branch of retroviruses that have an ISD and covalent TM (Figure 7). The group that includes GPDRV TM includes sequences from betaretroviruses and gammaretroviruses, but due to the relatively short length of the TM domain, branches of the tree generally had low support values. 

### 3.5. Integration Site Analysis

Identification of integration sites in tumor DNA resulted in four unique sites; two sites were identified in the 14-1342 tumor and two in the 15-1406 tumor. The Gunnison’s prairie dog genome sequence recently became available [47], which enabled identification of the genome location of the four integration sites. The GPDRV sequence in the 14-1342 tumor was found to be integrated at positions 309,469 (+ orientation) and 81,902 (– orientation) and the GPDRV sequence in the 15-1406 tumor was integrated at positions at positions 1,301,104 (+ orientation) and 629,910 (– orientation).

### 3.6. Screening for GPDRV with Virus-Specific Primers

Following the identification of two retroviral sequences with the metagenomics approach we developed virus specific primers to screen additional samples. We selected 42 prairie dog spleen samples to screen for the presence of ERV-PDRV.1-*Cynomys ludovicianus* using virus-specific primers (494F/591R) that amplify a region spanning *pro-pol*. This sequence was detected in DNA from all (*n* = 42) prairie dog spleen samples tested, strongly supporting this as an endogenous retroviral sequence. These samples represented Gunnison’s (*n* = 25), black-tailed (*n* = 14), and white-tailed (*n* = 2) prairie dogs.

Alignment of the endogenous and exogenous sequences was used to identify primers that specifically amplify only *gag* from the exogenous GPDRV. Of the 42 prairie dog spleen samples, GPDRV was only detected in the spleen (and tumor) from 14-1342 and 15-1406 and in the spleen of a Gunnison’s prairie dog (14-1344) without a tumor. Animal 14-1344 was from the same colony as 14-1342.

Spleen tissues from all 125 prairie dogs in this study were screened for GPDRV using *env*-specific primers 5729F/5840R (Table 1). GPDRV was detected only in Gunnison’s prairie dogs, including detection in thymus from 11-1310 and spleen and thymus from 14-1342 and 15-1406. We also detected GPDRV in spleen from four additional Gunnison’s prairie dogs that did not have tumors observed at necropsy. Causes of death in these four prairie dogs included: plague (16-633 and 16-778), tularemia (16-675), and suspected stress-related capture mortality (14-1344). Detection of GPDRV RNA is therefore significantly associated with presence of thymic lymphomas (Fisher’s exact test; *p* = 0.002).

Using primers located in U3 and *env* we were able to amplify a 7.14 kb fragment from the spleen of animal 14-1344. Sequencing of this product confirmed the presence of GPDRV with 99.7% and 99.1% nucleotide identity with the assembled genomes from 14-1342 and 15-1406, respectively.

## 4. Discussion

In any population, clusters of tumor cases can suggest an underlying or predisposing factor [48,49,50], and three cases of thymic lymphoma in Gunnison’s prairie dogs in Colorado, USA warranted further investigation. We specifically investigated a possible retroviral etiology due to similar retrovirus-associated lymphoid tumors in other species [11,14,15,51,52]. Using PCR and next generation sequencing, we identified and were able to assemble two unique retroviral sequences from lymphoid tissue of prairie dogs.

One of the sequences was identified in DNA from all samples screened, which included members from each of three species of prairie dogs included in the study. This widespread occurrence is consistent with the expected distribution of an endogenous viral sequence [45,53,54]. This sequence lacked an apparent *env*-coding region, suggesting a possible mutation/deletion typical of endogenous viruses [53,54]. We suspect that this consensus sequence likely represents endogenous retroviral sequence(s) of prairie dogs, and we propose the name ERV-PDRV.1-*Cynomys ludovicianus* [55]. The presence of ERV-PDRV.1-*Cynomys ludovicianus* in all three species of prairie dogs suggests that endogenization occurred prior to evolutionary divergence of these species [45,53,56]. Screening of other prairie dog species and other *Sciuridae* species may provide further insights as to when this viral sequence was acquired. Gifford et al. [45] screened for the presence of class II endogenous retroviral sequences using conserved PR and RT primers and identified a sequence from a black-tailed prairie dog that is 62% identical to the ERV-PDRV.1-*Cynomys ludovicianus* and 59% identical to GPDRV nucleotide sequences identified in this study.

A second retroviral sequence identified in thymic tissue and spleen of two prairie dogs with thymic lymphoma included *gag-pro-pol-env* coding regions. Using *env*-specific primers this sequence was identified in thymic tissue and spleen from all (3/3) of the prairie dogs with thymic lymphoma, and from splenic tissue of only 3.3% (4/122) of prairie dogs that did not have tumors identified grossly. All of the *env*-positive animals were from prairie dog colonies located in the Gunnison Basin in Colorado, USA. Gunnison’s prairie dogs located outside the Gunnison Basin were all negative for GPDRV. This consistent association with tumors and infrequent occurrence in the overall population of animals examined in the study is an expected pattern for an infectious, exogenous oncogenic virus. Isolation of virus in culture was not pursued in this study. The finding of multiple integration sites supports classification as an exogenous infectious virus. We suggest that this sequence represents the first exogenous retroviral sequence identified in prairie dogs and propose the name Gunnison’s prairie dog retrovirus (GPDRV).

As we were finalizing this paper, a Gunnison’s prairie dog genome assembly was published [47]. This assembly contained multiple contigs with sequences similar to both GPRDV and to ERV-PDRV.1-*Cynomys ludovicianus*. A BLASTN search of the assembly with ERV-PDRV.1-*Cynomys ludovicianus* yielded 3156 alignments with E-values <1e-10. Many of these are nearly identical to the ERV-PDRV.1 sequence over more or less its entire length: 652 of the alignments cover >80% of the ERV sequence with >90% identity. The highest scoring alignment was 95.9% identical over 100% of the ERV-PDRV.1 sequences, which represents a consensus sequence assembled from our metagenomic datasets. A similar BLAST search with GPDRV produced 759 alignments with *E*-values <10^−10^. For these, the highest scoring alignment was on 84.9% identical to GPDRV, over 99% of the GPDRV sequence. We conclude that the Gunnison’s prairie dog genome contains a large number of sequences related to both GPDRV and to ERV-PDRV.1 that may represent endogenized retrovirus sequences and possibly proviruses from other exogenous retroviruses.

The genetic organization of GPDRV is typical of a betaretrovirus. The protease and polymerase proteins are expected to be expressed as Gag-Pro and Gag-Pro-Pol polypeptides by ribosomal frameshifting. The protein encoded by *pro* harbors dUTPase and active protease domains. Phylogenetic analysis based on Pol amino acid sequences supported the classification of GPDRV as a betaretrovirus.

The retrovirus envelope protein is cleaved by cellular furin to generate SU and TM subunits. The SU and TM subunits remain associated after cleavage either through noncovalent interactions or formation of a covalent bond, which is determined by the cysteine motif located between the heptad repeats within TM [57,58]. The betaretroviruses, MMTV, JSRV and ENTV, contain a CX_7_C motif, which forms noncovalent interactions [59]. The TM subunit found in alpha-, gamma- and delta- retroviruses contain a cysteine motif, CX_6_CC that forms a covalent bond with a cysteine in the SU subunit [57,60,61,62]. The TM subunit of alpha-, gamma-, delta-retroviruses and the D-type betaretrovirus, Mason-Pfizer monkey virus (MPMV), also contain an immunosuppressive domain [46,63,64]. The envelope gene of MPMV was derived by a recombination event, which resulted in the acquisition of an envelope gene from a gammaretrovirus [46,65]. The GPDRV TM harbors a CX_6_CC and an immunosuppressive domain similar to that of MPMV suggesting GPDRV is a D-type betaretrovirus that may have undergone a similar recombination event. 

The significance of GPDRV to prairie dog populations is uncertain. For all three cases with thymic lymphoma, the tumor was determined to be the cause of death or related to the cause of death by compromising cardiovascular function under anesthesia. However, overall occurrence of thymic lymphoma was low (3/125) and not considered to be a significant source of mortality at the population level. Of the four prairie dogs which tested positive for GPDRV but were unaffected by thymic lymphoma, three died from bacterial infections. Based on the immunosuppressive effects of other retroviruses, we considered possible population effects due to increased susceptibility to bacterial infections. However, prairie dogs positive for GPDRV were not over-represented among animals that died from bacteremia. Although three of the seven (43%) prairie dogs with GPDRV died from bacterial infections, 51 of the 118 (43%) prairie dogs without GPDRV also died from bacterial infections. One prairie dog that was positive for GPRDV but unaffected by thymic lymphoma died from capture-related factors (heat stress) with no signs of immune suppression. This rare complication of trapping is unfortunate but GPDRV was not suspected to be associated with the cause of death in this animal. No other lesions observed were suggestive of immune suppression. The small proportion of animals testing positive for GPLDRV prevented further analysis of possible effects of the virus.

GPDRV is statistically associated with thymic lymphoma in Gunnison’s prairie dogs. Future investigations could include isolation of virus, experimental infection studies, and corroboration of the association with larger sample sets. Further analysis of integration sites may yield insight into potential mechanisms of oncogenesis. Surveillance areas were limited to the state of Colorado, with access to only three prairie dog species. GPDRV sequences were only found in Gunnison’s prairie dogs. This could suggest either species specificity or lack of exposure in other species. Continued surveillance of other prairie dog species may help understand the species host range of the virus.

## Figures and Tables

**Table 1:** PCR primers.

Primer Designation	Primer Sequence
*caf1*-F	5′ ATA CTG CAG ATG AAA AAA ATC AGT TCC 3′
*caf1*-R	5′ ATA AAG CTT TTA TTG GTT AGA TAC GGT 3′
P2	5′ TAG GAT CCC ATT AGC TGT CCA CTT ACC 3′
P3	5′ GGA ATT CGT TAG GTG GCT CTG ATG AT 3′
YMDD	5′ ATC AGA TCC TAC TAA CDR TCR TCC ATR TA 3′
LPQG	5′ TAC CAG TGG AAT GTT CTA CCN 3′
LTR-F	5′ GAC CGT GAC TTG TTT ATC TAA CCA CAA 3′
Env-7073	5′ AGA CTG CAA TCT TTG GTA ATG AAC CTG 3′
SP3	5′ AAG GTT CTT CAT CCA GGA GGT ATA TCT C 3′
SP4	5′ TTT GGA CTT CAA CCA TGG GGA AAA TAC 3′
SP5	5′ TTC CGT ACT TAC CCC TTC TTT CCG AT 3′
LTR-R	5’CCA AGG TTC TTC ATC CAG GAG GTA TAT 3′
494F	5′ AAG GAT GTG AAG GAA CTA TAC AGC CAT 3′
591R	5′ TCG GGG TGA ATT GGA ATT GAA AAG AAA 3′
393F	5′ ATGCTGACTGGGATATGGTCAAAAATG 3′
1961R	5′ GTATCGGGTTTCCTTGGACATCAAATT 3′
5729F	5′ GGATGGCAAATCGTATTATCAGGCTAC 3′
5840R	5′ TAGAGTTCCCACTGAGGTACCTAAGAT 3′

**Table 2:** Causes of death in 125 prairie dogs in Colorado from 2009 to 2018.

Cause of Death	#	G ^a^	BT ^b^	WT ^c^	TL ^d^	GPDRV ^e^
Thymic lymphoma only	2	2	0	0	2	2
Bacterial	54	21	23	10	0	3
Plague	49	20	19	10	0	2
Tularemia	2	1	1	0	0	1
Other bacteremia	3	0	3	0	0	0
Trauma	21	14	3	4	0	0
Capture related	10	9	1	0	1	2
Environmental	8	3	5	0	0	0
Intraspecific aggression	6	2	1	3	0	0
Euthanized for disease concern	4	1	3	0	0	0
Undetermined	20	7	4	9	0	0

^a^ G Gunnison’s prairie dog. ^b^ BT black-tailed prairie dog. ^c^ WT white-tailed prairie dog. ^d^ TL thymic lymphoma. ^e^ GPDRV Gunnison’s prairie dog retrovirus.
